# A simulation-based hybrid causal predictive framework for stockout risk analysis in supply chain

**DOI:** 10.1371/journal.pone.0350429

**Published:** 2026-06-11

**Authors:** Emad Hafaf, Ahmad Bassam Alzubi, Kolawole Iyiola, Hasan Yousef Aljuhmani

**Affiliations:** Institute of Graduate Research and Studies, University of Mediterranean Karpasia, Mersin, Turkey; Istinye University: Istinye Universitesi, TÜRKIYE

## Abstract

Stockout risk is a persistent challenge in supply chain management, undermining both operational efficiency and customer satisfaction. This study adopts a multi-method approach to investigate the causal effect of lead time on stockout risk by integrating causal inference techniques with predictive analytics. The proposed framework combines Propensity Score Matching (PSM), Instrumental Variables (IV-2SLS), Inverse Probability Weighting (IPW), and Doubly Robust Estimation (DRE) alongside machine learning (ML) algorithms and time series forecasting. Using a dataset of 20,000 supply chain incidents, the study estimates the Average Treatment Effect (ATE) and evaluates predictive model performance. PSM generated the most credible ATE (0.882), confirming a strong causal link between lead time and stockout risk. IV analysis using supplier distance as an instrument yielded a reduced and statistically insignificant ATE (0.5535, p = 0.3148), suggesting instrument weakness. Among ML models, Random Forest and LightGBM achieved superior predictive accuracy (R^2^ = 0.25; MSE = 0.736), while Moving Average forecasting effectively captured stockout patterns over time (R^2^ = 0.883). The findings identify PSM as the most robust technique for causal inference. This study advances the literature by integrating causal inference, ML, and time series methods, offering practical, data-driven insights to strengthen operational resilience and guide proactive inventory management.

## 1. Introduction

In the evolving world, Supply chain management has adopted more sophisticated approaches to reduce risks and improve operational services [1]. Notably, Data analysis and Machine learning (ML) have been used to enhance this such as in, supplier risk evaluation, stock optimization, and demand forecasting. Hence, by comparing these different databases to find trends and anticipate threats, these methods have shown promise in improving supply chain resilience [2]. Therefore, methods for causal ML have been proposed for creating supply chain risk management response [3]. Recent studies have increasingly concentrated on applying novel methods to predict and minimize the risks related to late deliveries and stock outs.

**Fig 1:**
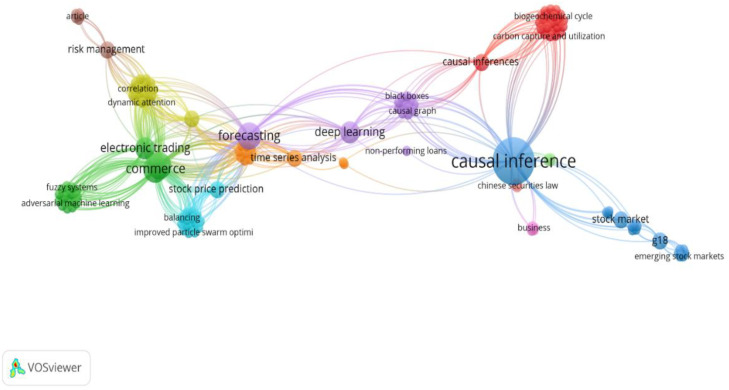
Bibliometric report [40].

Furthermore, Artificial intelligence (AI) and ML are sophisticated intelligent tools transforming the supply chain management sector, by allowing for data-driven decision-making, optimizing processes, and improving performance across the supply chain network [4]. With a rapid increase of data from numerous sources, such as Internet of Things (IoT) devices, enterprise resource planning (ERP) systems, transportation systems, and customer demand information, AI and ML approaches have increasingly become vital in handling the multifaceted nature of contemporary supply chains [5]. These tools aid in demand prediction, where predictive algorithms use previous sales data, seasonal trends, and other variables like economic patterns and atmospheric conditions to effectively estimate projected demand [6]. This enables businesses to optimize inventory levels, prevent stockouts, and avoid overstock situations, which can lead to higher carrying costs. AI-driven inventory optimization allows for tracking and supply, decreasing waste and improving supply chain efficiency [7]. Fig 1 illustrates the bibliometric report on the paper review on Scopus. Over 22 published articles were identified using keywords such as supply chains, ML, prediction and causal inference between 2024 and 2025. The bibliometric visualization was done using the VOS viewer application as shown in Fig 1.

AI-influenced forecasting and other risk factors conditions enable different businesses to identify and curtail possible supply chains issues before they occur. Thus, this process improves procurement, supplier risk control, logistics control through applying techniques like the ML, Deep Learning and reinforcement learning [8].

Additionally, a more flexible, robust and cost-effective supply chain that adapts to changing in both demand and supply factors are integrating AI in analysis and reduces risks and boosts customer satisfaction [9]. Another important use of AI in supply chains is risk management, particularly in managing stockout problems. ML algorithms can analyze vast datasets to identify trends and predict crises prior to the occurrence, allowing businesses to take preemptive risk management measures. Businesses that use AI-driven analytics may be able to establish a more responsive, flexible, and cost-effective supply chain that reacts to changing demand and supply scenarios, reducing uncertainty and improving customer satisfaction [10].

Comparatively, Causal inference is an imperative analytical tool utilized in supply chain that assists in decision-making to distinguish between correlation and causation for evaluation of variables which influences the stockouts risk [11]. Comparatively, traditional statistical methods and ML models often focus on the accuracy in prediction but fail to discover the fundamental causal relationships between supply chain components. For example, while an algorithm could demonstrate how longer lead times are related with increased stockout risk, it cannot demonstrate that decreasing lead time will really reduce stockouts [12]. Causal inference has grown increasingly significant in supply chain management because it allows organizations to go over correlation-based observations and discover actual cause-and-effect linkages. Unlike conventional predictive analysis, which focusses on projecting future trends, causal inference approaches can assess the direct influence of variables such as lead time, supplier disruptions, price strategies, and demand changes on stockout risk and final supply chain performance [13].

To address these research gaps and achieve a comprehensive understanding of stockout risk, this study is guided by the following key objectives:

1. To develop a hybrid decision-layered framework that combines causal inference, predictive modeling and temporal analysis within a unified architecture for stockout risk assessment.
2. To validate the integrated framework in a simulation-based environment, enabling controlled methodological evaluation of causal and predictive components.
3. To evaluate intervention relevance through estimation of the causal effect of the lead time on stockout risk using established causal inference methods under explicit identification assumptions.
4. To support operational forecasting by implementing ML methods for short-term prediction of stockout risk based on multivariate supply chain features.

## 2. Related studies

Modern studies have focused on risk assessment and mitigation in supply chains, using a novel analytical tool such as ML, causal inference and Causal ML, and time series forecasting. Therefore, this section combines previous work into three major topics and identifies specific gaps that drive our harmonious, multi-method framework.

### 2.1. Predictive ML approach in supply chains

Predictive modelling in supply networks is an important analytical strategy that utilizes ML and sophisticated mathematical methods to forecast future supply chain behaviors, optimize resource allocation, and reduce risks associated with unpredictable demand and logistical uncertainty.

[14] adopted deep learning algorithms in macroeconomic statistics to predict late deliveries in the automobile industry, emphasizing the importance of external economic problems on supply chain efficiency. Additionally, in a predictive study by [4] that evaluated the efficacy of ML techniques for forecasting customer backorders to enhance the efficiency in supply chain. The study focusses on the difficulty of balancing accurate models and computational effectiveness in complicated supply chain contexts. By comparing the approaches generated with 22 variables to only the best five, the authors showed that simplified ML algorithms can dramatically cut computational expenses by 30% to 98%, while maintaining good accuracy with 0.6% to 4.2% decrease in F1-score. Applying accessible datasets, the study demonstrates the practical benefit of simplified prediction algorithms for real-time inventory and backorder management, indicating their suitability for wider industrial deployment.

In a similar study, [15] employed ML and AI approaches to forecast delays in truck delivery operations. Their research tackles transportation management issues by predicting delays, allowing management to more accurately plan and deploy resources. Practical logistical data was analyzed using a variety of ML algorithms, such as decision trees, random forests, Artificial Neural Networks (ANN), and support vector machines. The study revealed that each of these algorithms predicted delays with up to 97.6% accuracy, with the Adaboost classifier beating all of them in both accuracy and precision. This study illustrates the possibility of ML to improve logistics efficiency and reduce supply chain delays [2,15] examined the application of ML approaches to prediction in supply chain networks. The study addresses the constraints of traditional maintenance techniques by using ML methods to detect equipment failures, reduce unexpected downtime, and improve supply chain efficiency. By analyzing external variables, sensor inputs, and historical data, the research reveals how predictive analysis may detect possible issues before they cause disruptions. The study compares multiple ML models designed for maintenance prediction, providing useful insights into how ML may optimize supply chain operations and increase efficiency through proactive maintenance tactics [15].

A comprehensive study by [16] reported the development of a ML-based predictive and alerting system to improve practical decision-making system in a computerized warehouse setting. Their algorithm forecasts identification of late order using information gathered by Warehouse Management Systems (WMS), allowing for action through the designed alerting setting. Similarly, a tyre distribution company adopted this technology to validate a Shuttle-Based Storing and Recovery system. The approach successfully adopted some approaches by evaluating several ML models and customizing predictions for warehouse-specific factors. Their study focuses on the practical advantages of ML for improving short-term operational effectiveness in logistics [16].

In a recent study by [17] shows how hybridization and improved integration of models could enhance prediction. The study presented a priority-based architecture integrating space-time analysis and also a crash severity technique to assess pedestrian and bicyclist safety. Their results show the imperativeness of hybridization in prediction and how it influences outcome.

### 2.2. Causal inference and causal ML in supply chains

Causal inference methods have advanced beyond just understanding the relationships between variables to identify causal frameworks, but estimate treatment effects, forecast counterfactual events, and guide decision-making procedures in complicated real-world systems. Studies by [3] made a proposal for the implementation of causal ML to create intervention techniques, emphasizing the importance of understanding relationships between variables in order to improve decision-making. Similarly, [18] presented a novel method integrating causal discoveries with reinforcement learning to ascribe the fundamental causes of supply risks, tackling the complications of interdependence in supply chains. In the medical supply chains sector, [19] also reported two-stage methodologies integrating both ML and a Unified Robust Stochastic Programming (URSP) architecture to improve the survivability of medical supply chains (MSCs) during disease outbreaks globally. The first phase employs an ML algorithm to forecast infection levels and change demand projections accurately. To handle constraints under unpredictability, on the other hand, the second stage employs URSP, which combines stochastic programming and robust optimization with a configurable strength level, as well as Conditional Value-at-Risk (CVaR). This case study in Turkey showed that the integrative strategy outperformed previous methods by providing more cost-effective and robust allocation options. The study emphasizes the significance of combining predictive modelling and robust decision-making techniques to overcome substantial ambiguity within the healthcare supply chain [19].

While causal techniques can help with hypothetical intervention planning and identifying supply chain risk factors, new research shows that these methods are not enough to back up operational decision support in its entirety, since they cannot reliably predict the short term or analyze temporal patterns. In their discussion of the shortcomings of models that attempt to infer causation alone, [20] highlight the necessity for hybrid analytical techniques that also incorporate predictive performance and planning skills.

### 2.3. Hybridization of intelligent computational systems

The adoption of multiple intelligent mathematical models in supply chains as an advanced approach that integrates various computational frameworks to address various supply chains decision problems resulting in significantly robust and responsive supply chain systems. Many research studies have reported the hybridization [21] of these multiple models in addressing supply chains, such as [5] who assessed the application of intelligent computational methods such as IoT, ML, AI, and blockchain to improve accuracy and openness in smart manufacturing supply chains. The study focusses on two application cases, demand-side forecasting and buyer.

Furthermore, the increasing complexities in supply chain networks have resulted to better reliance on predictive models and causal inferences to minimize stockout risk. Recent studies have shown a various way to improve risk management, known causal ML, reinforcement learning and Bayesian networks [22]. Causal inference has grown increasingly significant in supply chain management because it allows organizations to go over correlation-based observations and discover actual cause-and-effect linkages. Unlike conventional predictive analysis, which focusses on projecting future trends, causal inference approaches can assess the direct influence of variables such as lead time, supplier disruptions, price strategies, and demand changes on stockout risk and final supply chain performance [23]. Table 1 shows the summary of some related studies that adopted various ML techniques.

**Table 1:** Summary of some related studies.

S/N	Study	Method	Reference	Aim	AI-technique
1	Causal ML in supply chains Risk management	Applying causal ML approaches to create strategies for supply chain risk management.	[3]	To study maritime engineering dataset on how causal machine learning (ML) improves decision-making by reducing risks.	CML
2	Root Cause Attribution of Delivery Risks via Causal Discovery with Reinforcement Learning	The study incorporates reinforcement learning in conjunction with causal research to determine the underlying reasons behind delivery delays.	[18]	To provide a roadmap for proactive initiatives to reduce delivery risks by prioritizing influential links in order to quantify causal strengths.	Reinforcement Learning
3	Maximizing supply chain performance leveraging machine learning to anticipate customer backorders	Simplified ML techniques for backorder prediction	[4]	To enhance customer backorder prediction using basic ML models.	ML Models (Random Forest, SVM, ANN, and Decision Trees)
4	Smart manufacturing supply chain process strategy using intelligent computation techniques	Blockchain for Product Traceability, IoT, ML, and	[5]	To increase openness and traceability in production systems.	IoT, ML (Predictive Algorithms) and Blockchain
5.	Predicting Delays for Truck Delivery Logistics: An Application of AI and ML	Predictive ML for Forecasting Logistics Delay	[15]	To use ML to forecast logistics delays in truck deliveries.	ML models such as ANN, Random Forest, SVM, and Adaboost)
6.	Scopus – Document details – Leveraging Machine Learning for Predictive Maintenance in Supply Chain Management Systems | Signed in	Prediction Maintenance in SCM using ML.	[2]	Use ML to forecast equipment breakdowns in supply chain systems.	ML Forecast and Failure Detection
7.	A comprehensive methodology combining machine learning and unified robust stochastic programming for medical supply chain viability	ML, Robust Optimization (URSP)	[19]	ML and URSP to ensure the healthcare supply chain’s viability	To improve the efficiency of the healthcare supply chain utilizing machine learning and URSP.
8.	Making in Warehouses: A machine learning-based forecasting and alerting system for cycle time prediction	Prediction and warning system based on ML	[16]	ML Modelling for Prediction and Forecasting	To estimate operational interruptions and warehouse cycle times.

Despite these advances in the various studies reported, a significant gap remains in merging causal inference approaches with predictive modelling to thoroughly assess and predict stockout risks. While ML algorithms have showed promise for detecting possible disruptions, they frequently fail to identify a causal relationship between significant factors like lead time and stockout risk. This constraint impedes the creation of efficient intervention techniques for reducing stockout incidents.

Our study addresses a research gap by combining causal inference and predictive modelling to understand and forecast stockout threats. While previous research focusses on either ML for forecasting or optimization approaches within certain contexts, it falls short of identifying the causal associations between major risks in the supply chain, like lead time consequences on stockouts.

### 2.4. Methodological contribution and framework synthesis

While many articles have applied numerous methodological frameworks such as, ML, econometric or time series techniques to supply chain risk issues, these approaches are consistently adopted in isolation and target a single analytical objective. Predictive models focus mainly on forecasting accuracy, time series approaches are focused on analyzing temporal behavior and causal inference focuses on intervention effects. However, the operational decision-making necessitates the coherent knowledge on potential outcomes such as, when risk is likely to occur, what drives disruptions and how risk evolves with time. Therefore, lack of structured integration of these factors draw the practical value of current analytical techniques.

This study, contributes methodologically by proposing a simulation-Based Hybrid causal-predictive framework that establishes the integrated functionality of predictive modeling, causal inference and temporal analysis into a single decision-oriented architecture. Hence, the contribution does not involve proposing new estimators, but in defining a structured synthesis that includes each analytical process serving a distinct and adjunct decision role.

## 3. Materials and methods

### 3.1. Research methodology

The study utilizes a simulation-based methodological analysis design to evaluate a hybrid decision-layered framework for stockout risk analysis. The methodological framework integrates causal inference, predictive modeling and temporal analysis within a structured architecture to intervene on relevance, operational forecasting and temporal monitoring.

**Fig 2:**
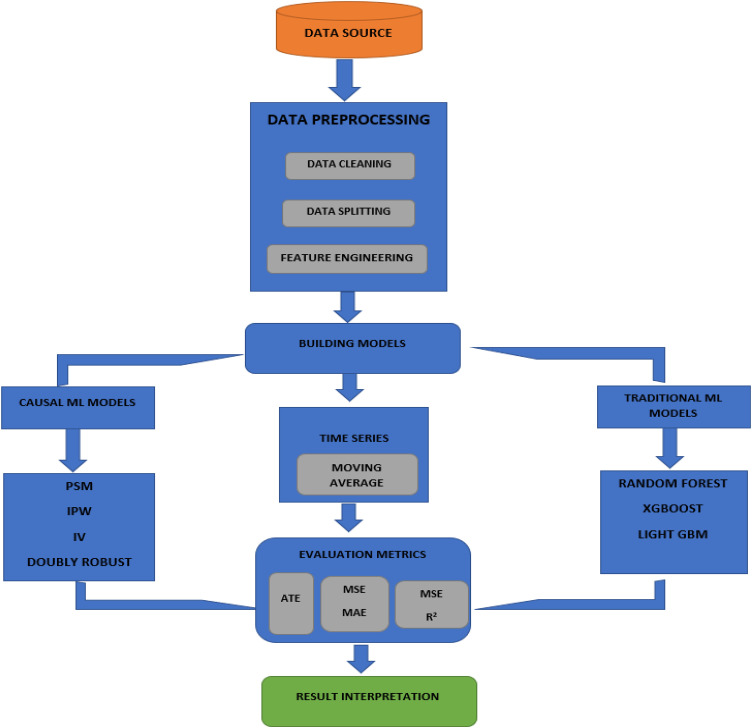
Experimental framework.

Furthermore, the methodological objective focuses mainly on the validation of the proposed hybrid framework under controlled and structured conditions. Therefore, a synthetic dataset representing various supply chain operations based on similar studies, was generated to mimic realistic relationships within standard supply chains criteria such as, inventory levels, logistic factors, demand, stockout risk and supplier reliability. The simulation environment allows evaluation of analytical components characterized by well-defined dependencies and reproducibility. Data preprocessing steps such as Data cleaning, Data splitting and feature Engineering similar to [24] were adopted. Traditional ML models, causal inference techniques and time series were evaluated based on their evaluation metrics as shown in Fig 2.

The analytical elements that comprise the framework are assessed using different criteria since they perform diverse decision-making roles. The interpretability, strength across estimators, and conceptual validity of causal inference approaches are evaluated in relation to their ability to estimate intervention impact and their adherence to identification principles. When evaluating predictive models, statistical performance indicators such as MAE, MSE, RMSE, and R^2^ are used to gauge the accuracy of the forecasts. How well time-series approaches reflect the time frame and align their forecasts are two key metrics for evaluating them.

### 3.2. Implementation and platform

The experiment was carried out on Python (v3.9) using scikit-learn, causal ML, and other additional libraries. The analysis was carried out using python programming language. The Exploratory Data Analysis technique was adopted using normalization approach similar to study [25] where the dataset was standardized using the Minmaxscaler approach. Additionally, categorical dataset “supplier_type” was converted to numerical dataset using the OneHotEncorder approach.

### 3.3. Data description

The study adopted a synthetically generated supply chain dataset, structured to approximate real-world distribution network dynamics [26]. The dataset was generated using a parameterized probabilistic distribution, that reflects operational ranges in supply chains studies. The dataset contains 19 essential variables with 20,000 instances, relevant to supply chain operations. The variables include, order processing, inventory management, supplier reliability, logistics, and stockout risk factors. It provides integer-type variables like order_id, lead_time, inventory_level, demand, order_size, and warehouse_capacity to measure supply chain activities. Float-type characteristics such as supplier_reliability, stockout_risk, seasonality_factor, economic_index, warehouse_utilization, and market_demand_trend reflect ongoing risks and economic impacts. Additionally, the supplier_type feature represents categorical data. These characteristics together form a solid foundation for analyzing stockout risk and optimizing supply chain robustness through causal inference, machine learning, and time series forecasting. The data is briefly summarized on Table 2.

**Table 2:** Data description.

Variable	Unique values	Data type	Sample values
Lead time	25	Int 64	11,24,19
Order_id	20000	Int 64	1,2,3
Inventory_level	4875	Int 64	238944062146
Supplier reliability	20000	Float 64	0.559, 0.67, 0.74
Demand	980	Int 64	195439751

### 3.4. Causal inference

In the causal analysis executed in the study, the treatment variable was clearly stated as prolonged lead time, operated as a binary indicator. For every observation *i*∊ {1,2, …..,N} the treatment assignment *T*_*i*_ is specified as;


$$\begin{array}{c} Ti=\text{1},ifLi>\text{median }(\text{L}) \end{array}$$

$$\begin{array}{c} Ti=\text{0},ifLi\leq \text{median }(\text{L}) \end{array}$$ (1)

Where;

Li represents the lead time for observation i while L symbolizes the collection of lead-time values in the dataset. The result variable Yi∈[0,1] signifies stockout risk, characterized as a normalized probability of stockout incidence under operational conditions.

Where; *i*∈ {1,2,…,N} indexes the observations, Tᵢ ∈ {0,1} represents treatment indicator, and Xᵢ ∈ ℝᵖ is the vector of observed covariates.

Causal inference methods are necessary to go beyond relationships and comprehend the actual impact of lead time on stockout risk. In contrast to conventional ML algorithms that only concentrate on prediction, causal inference approaches assist supply chain managers adopt data-driven policy decisions to lower stockout risk by identifying cause-and-effect linkages [23].

**Fig 3:**
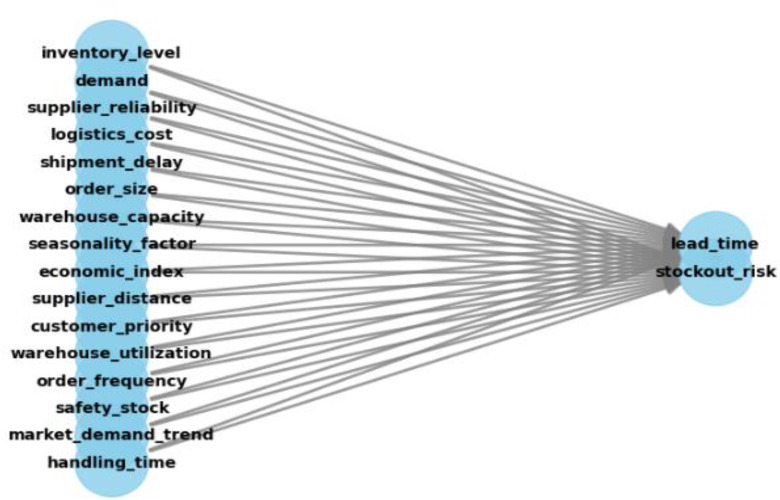
Causal graph.

The relationships between numerous supply chain elements and two crucial variables, lead time and stockout risk, are shown in Fig 3. Numerous predictors, including inventory level, demand, supplier dependability, logistics costs, shipment delays, and warehouse capacity, are shown on the left side. These elements affect the likelihood of a stockout and the operational effectiveness of the supply chain. Thus, the pointed arrows indicate these independent parameters possible impact on lead time and stockout risk by illuminating the causal and predictive connections between them and the dependent parameters. Similar to research by [27], this visualization helps the study achieve its objective of better explaining and forecasting stockout difficulties in supply chains through the use of causal inference, ML, and time series forecast.

The causal graph in Fig 3 shows how 17 important supply chain factors, including demand, inventory level, logistics cost, and supplier dependability, directly affect two important outcomes: lead time and stock out risk. A putative causal relationship is represented by each arrow, emphasizing the ways in which operational, logistical, and economic variables influence these targets’ variability. In order to maximize supply chain performance, this framework facilitates intervention planning and predictive modelling.

The causal inference method for the suggested strategy assesses the impact of lead time on stockout risk based on a number of conventional identification assumptions. These assumptions describe the circumstances in which the predicted Average Treatment Effects (ATEs) may be evaluated logically instead of associatively. It is considered that all pertinent confounders influencing both lead time and stockout risk are accounted for conditional exchangeability, that units possess a non-zero likelihood of being assigned either of the treatment and control groups and that every single supply chain incident is distinct with no influence among observations. In the case of instrumental variable assessment, the instrument is presumed to be associated with lead time and to influence stockout risk solely through this mechanism. These hypotheses direct the assessment of causal estimates in the simulated framework.

#### 3.4.1. Propensity Score Matching (PSM).

PSM is a technique that uses a quasi-randomized test to determine the causal relationship between lead time and stockout risk. It compares observations that have been treated and those that have not, according to the propensity score that indicates the probability of receiving the treatment.


$$\begin{array}{c} \text{e}({\text{X}}_{\text{i}})=\text{P}({\text{T}}_{\text{i}}=\text{1}|{\text{X}}_{\text{i}}) \end{array}$$ (2)

where;

e(Xᵢ) shows the probability that observation *i* is assigned the treatment based on the observed covariates *Xᵢ* where *i ∊ {1,2,…,N}* indexes the data observations, *Ti ∊ {0,1}* signifies the treatment indicator, and *Xi ∊ R*^*p*^ signifies the vector of p observed covariates [28].

#### 3.4.2. Doubly robust estimation.

Doubly robust approaches integrate IPW and regression modifications to produce consistent results regardless of whether one of the models is incorrectly defined [1].

The doubly robust estimator is defined as:


$$\begin{array}{c} \text{ATE }=\left(\frac{\text{1}}{\text{N}}\right)\sum \left[\frac{Ti\left(Yi-gi(Xi)\right)}{e(Xi)}-\frac{(\text{1}-Ti)\left(Yi-go(Xi)\right)}{\text{1}-e(Xi)}+g\text{1}(Xi)-go(Xi)\right]\text{P}({\text{T}}_{\text{i}}=\text{1}|{\text{X}}_{\text{i}}) \end{array}$$ (3)

Where;

*i ∊ {1, 2,…, N}* indexes the observations, *N* is the total number of observations, *Ti ∊ {0,1}* is the treatment indicator, *Yi* denotes the stockout risk, *Xi ∊ R*^*p*^ represents the vector of observed covariates, e(*Xi*) represents the propensity score, and *g₁(Xᵢ)* and g*₀(Xᵢ*) are outcome models for treated and control categories [29].

#### 3.4.3. Instrumental Variables (IV).

Instrumental Variables (IV) analysis is employed whenever the assignment of treatment is intrinsic, which means it is influenced by unobserved variables that impact the final result. Supplier distance is used as an instrumental variable for lead time, believing that it exclusively influences stockout risk by lead time.


$$\begin{array}{c} Ti=\alpha \text{0}+\alpha \text{1}Zi+\alpha \text{2}Xi+\in i \end{array}$$ (4)

Second-stage model:


$$\begin{array}{c} Yi=\beta \text{0}+\beta \text{1}Ti+\beta \text{2}Xi+ui \end{array}$$ (5)

Where;

*i ∊ {1,2,…,N}* represents the index observations, *Ti* denotes the treatment variable, which is the lead time, T_i_ is the predicted treatment from the first-stage model, *Yi,* denoting stockout risk, *Zi* represents supplier distance and *Xi* is the observed covariates [30].

#### 3.4.4. Inverse Probability Weighting (IPW).

IPW weights observations depending on their propensity scores, resulting in a reweighted pseudo-population that simulates a randomized experiment.


$$\begin{array}{c} wi=\frac{Ti}{e(Xi)}+\frac{\left(\text{1}-Ti\right)}{\text{1}-e(Xi)} \end{array}$$ (6)

Where;

*i ∊ {1,2,…,N}* indexes the observations, *wi* is the weight assigned to observation *i, Ti ∊ {0,1*} is the treatment indicator, and *e(Xi)* represents the propensity score, which is defined as the probability of receiving treatment given the observed covariates *Xi* [31].

### 3.5. Predictive modeling in supply chains

Predictive modelling is an essential framework in supply chain management that allows businesses to forecast stockouts, demand variations, and logistical problems. Predictive techniques employ ML techniques to analyze previous data, detecting trends and forecasting future events, resulting in enhanced decision-making and operational effectiveness [32].

#### 3.5.1. Random Forest.

Random Forest (RF) is an ensembled ML technique that builds numerous decisions trees and then combines their predictions to increase accuracy and avoid overfitting. It is especially beneficial for supply chain risk modelling since it accommodates non-linear interactions, performs effectively with high-dimensional dataset, and delivers significant feature insights [33].

RF classifier is made up of several decision trees *(T*_*1*_*, T*_*2*_*....., T*_*B*_), all are trained on a randomly chosen subset of attributes and a random portion of the data employing bootstrapping, also known as bagging. By combining each of the tree outputs, majority decision for categorization and average for regression, the ultimate prediction is produced [24].

**Step 1:** Bootstrapping and Feature Selection [34]


$$\begin{array}{c} \text{For every decision tree T}b\;(b=\text{1},\text{2}..,B) \end{array}$$ (7)

A randomly selected portion of the initial data (*D*_b_) is selected with substitution from the entire dataset(*D*).

At every divided a randomized subset of 𝑚 variables is chosen from the entire 𝑝 features (𝑚 ≪ 𝑝).


$$\begin{array}{c} f(x)=\text{1}/B\sum_{b=\text{1}}^{B}\text{Tb}(\text{x}) \end{array}$$ (8)

Where;

*f(x*)=predicted stockout risk for x input

*B* = number of trees.

*Tb(x*)= predicted from the both tree [35].

#### 3.5.2. Extreme gradient boosting for stockout risk prediction.

Extreme Gradient Boosting is a more advanced variant of gradient boosting which enhances accuracy of predictions, computing efficiency, and handling missing data. It is often employed in supply chain risk modelling because of its capacity to capture complicated interactions between variables while reducing overfitting through regularization.


$$\begin{array}{c} yi=\sum_{t=\text{1}}^{T}\text{ft}(\text{xi}) \end{array}$$ (9)

XGBoost creates an ensemble of additives decision trees, with each tree correcting its predecessor’s flaws by utilizing gradient descent to minimize a loss function [36].

#### 3.5.3. Light gradient boosting machine for stockout risk prediction.

LightGBM (LGBM) is a gradient boosting system that focusses on speed and efficiency in huge datasets. It varies from typical gradient boost approaches like XGBoost in that it employs statistical illustration-based learning and leaf-wise tree development, which makes it computationally efficient and scalable.

Similar to XGBoost, LightGBM iteratively optimizes the loss function by constructing an ensemble of decision trees. Nonetheless, it brings about significant advancements in data processing and tree growth.


$$\begin{array}{c} L(\theta )=\sum_{i=\text{1}}^{n}\text{ l}(\text{yi},\text{yi})+\sum_{i=\text{1}}^{n}\Omega (\text{fk}) \end{array}$$ (10)

Where; [37], L (yi, yi) is the loss function, Ω(fk) is used to prevent overfitting.

### 3.6. Time series forecasting

Time series forecasting is an important part of supply chain management because it allows firms to forecast prospective stockout risks using past trends. In contrast to traditional predictive modelling, time series approaches expressly consider variations in time in data, allowing organizations to foresee and manage risks. In this work, we adopted the Moving Average (MA) technique to evaluate and anticipate stockout risks [38].

#### 3.6.1. Moving average (MA) model.

The Moving Average (MA) approach is a basic time series method that smoothers variations in stockout risk by combining previous observations across a defined window. This strategy aids in the identification of patterns and seasonality, allowing for more informed inventory management decisions.


$$\begin{array}{c} Yt=\mu +\sum_{i=\text{1}}^{q}\theta i\in t-i+\in t \end{array}$$ (11)

Where; q represents the order of the moving average process, ∊ t is the white noise error, μ represents the mean of the series, *Yt* is the stockout risk [39].

### 3.7. Model evaluation

Performance metrics or model evaluation are quantitative evaluations of a model’s efficiency, particularly in mathematical modelling, ML, and prediction analysis. In regard to the task for which a model is intended, they assist both scholars and practitioners in evaluating the model’s general efficacy, precision, accuracy, and resilience [33]. In this study, the prediction algorithms’ accuracy and reliability were assessed using conventional performance indicators such as Mean Absolute Error (MAE), Root Mean Squared Error (RMSE), Mean Squared Error (MSE), and Coefficient of Determination (R^2^) as shown in Table 3.

**Table 3:** Performance Evaluation.

Metric	Formula	Range
MAE	$\text{MAE}=\frac{\text{1}}{N}\sum_{i=\text{1}}^{N}\left|Y_{obsi}-Y_{comi}\right|$	(0 < MAE < ∞)
MSE	$\text{MSE}=\frac{\text{1}}{N}\sum_{i=\text{1}}^{N}(Y_{obsi}-Y_{comi}{)}^{\text{2}}$	(0 < MSE < ∞)
R^2^	$\text{R2}=\text{1}-\frac{\sum_{j=\text{1}}^{N}{\left[{\left(Y\right)}_{obs,j}-{\left(Y\right)}_{com,j}\right]}^{\text{2}}}{\sum_{j=\text{1}}^{N}{\left[{\left(Y\right)}_{obs,j}-{\overset{―}{\left(Y\right)}}_{obs,j}\right]}^{\text{2}}}$	(0 < R^2^ < 1)
ATE	ATE=E[Y(1)−Y(0)]	- ∞< ATE < ∞

## 4. Results and discussion

### 4.1. Exploratory and dependency

The datasets were analyzed to find relationships between confounders and lead time as treatment variables to explore the interactions within the dataset.

**Fig 4:**
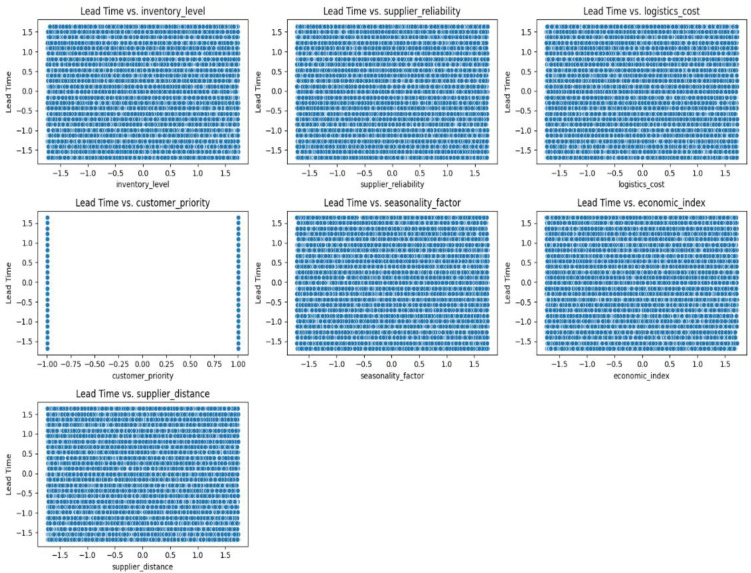
Correlation Scatter plots.

The scatterplots demonstrate an association between lead time and numerous supply chain variables. The data indicates extremely crowded and consistently distributed, indicating little relationship between lead time and the independent parameters. Additionally, client priority has a discontinuous distribution, which indicates categorical data. The visualizations point to the necessity for additional statistical evaluation, such as coefficients of correlation or a regression simulation, to evaluate potential correlations s shown in Fig 4.

**Fig 5:**
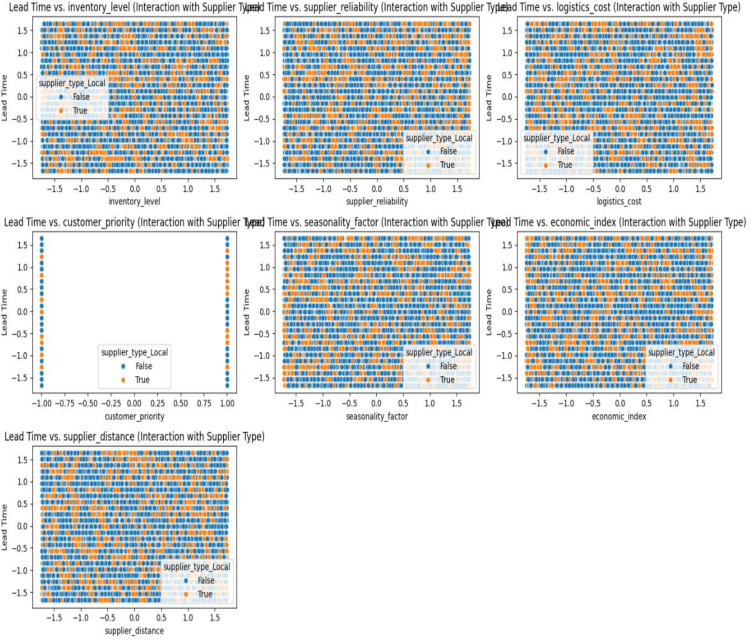
Scatter plots of interaction amongst the variables on lead time.

Furthermore, the scatter plots in Fig 5 demonstrate the interactions among numerous supply chain variables and supplier type on lead times. The visualizations indicate modest variations in lead time behavior depending on whether the provider is local or not.

**Fig 6:**
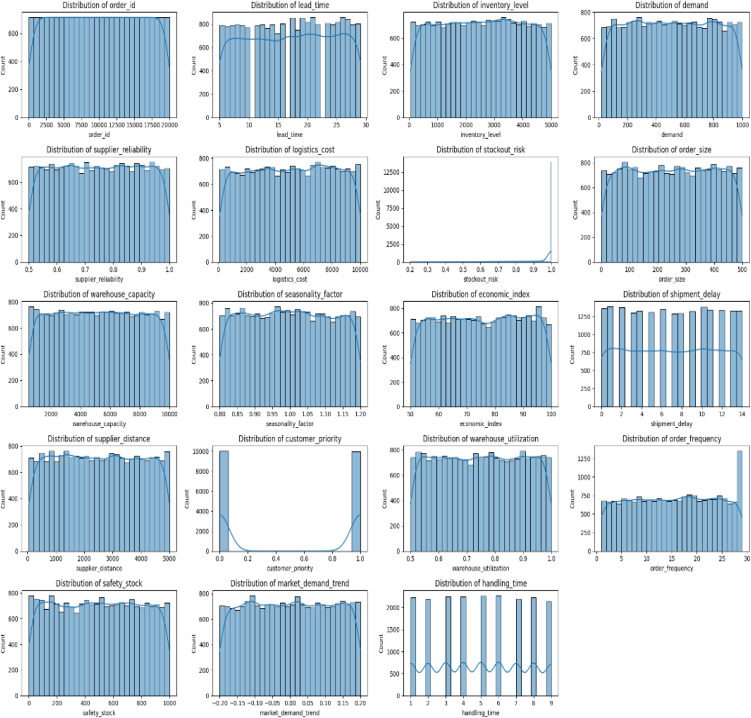
Distribution Plot.

The distribution plots in Fig 6 demonstrate variations in and distribution trends among major supply chain elements. Most numerical characters, including lead time, inventory level, demand, logistics cost, and supplier reliability, have a very equal distribution, signifying a harmonious dataset. However, stockout risk looks heavily skewed, implying that extreme outcomes (high stockout risk) are uncommon. Customer preference is bimodal, meaning two separate types of clients. Managing time and shipment delays exhibit different periodic patterns, which could imply systematic programming or regular influences. These findings inform feature selection and modification for predictive modelling and causal inference.

**Fig 7:**
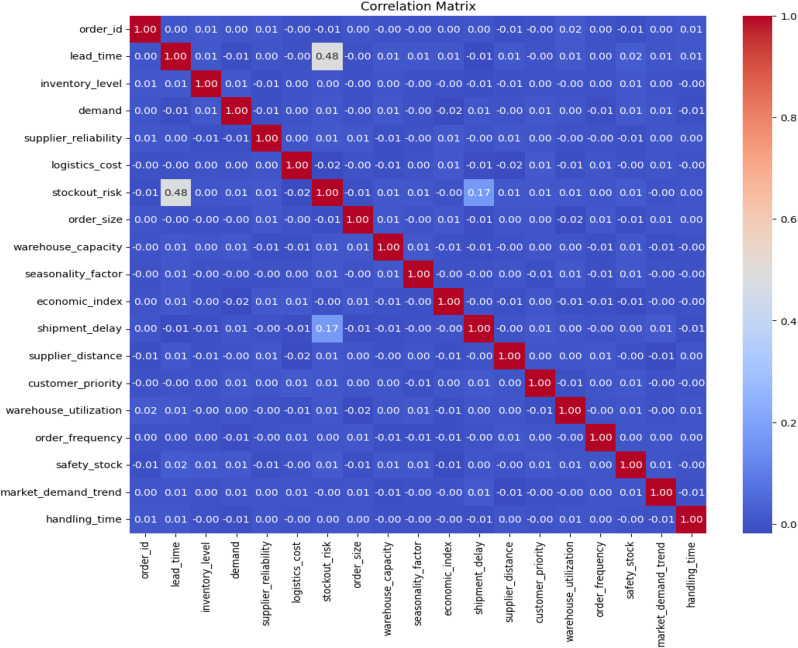
Correlation Matrix.

The correlation matrix represents the interactions among different supply chain parameters, emphasizing the pairwise dependencies. Lead time and stockout risk have a moderate positive connection (~0.48), suggesting that longer lead periods may increase stockout risks. Shipment delay has a smaller connection (~0.17) with stockout risk, indicating a possible but indirect influence. The majority of other features have low correlations, indicating that there are few direct linkages. This insight improves feature selection for predictive modelling and causal inference by focusing on the most pertinent factors influencing stockout risk as shown in Fig 7.

### 4.2. Experimental results

Table 4 shows how the study analyses stockout risk thoroughly by combining causal inference approaches, ML techniques, and time series forecasting. Amongst the causal inference approaches, PSM and IPW showed substantial ATE, demonstrating a strong causative association between lead time and stockout risk. The IV technique however, had a lower ATE of 0.5535, with a P-value of 0.3148 and a R^2^f 0.2435. This suggests a weaker causal effect, possibly due to poor instrument selection or unknown confounders.

**Table 4:** Comparative results.

Method	Model	ATE	P-Value	R^2^	MSE	MAE
**Causal Inference**	PSM	0.882	–	–	–	–
	IPW	0.864	–	–	–	–
	IV	0.5535	0.3148	0.2435	–	–
**Predictive Modeling**	RF	–	–	0.25	0.736	–
	XGBM	–	–	0.153	0.835	–
	LGBM	–	–	0.25	0.741	–
**Time Series Forecasting**	Moving Average	–	–	0.883	0.781	0.675
**Benchmark Model**	Linear regression	–	–	0.00063	18.89	–

RF and LGBM generated a R^2^ of 0.25, but XGBM had a lesser R^2^ of 0.153 but an increased MSE of 0.835, indicating differences in predicting accuracy. The Moving Average technique excelled other approaches with the greatest R^2^f 0.883 and the lowest MAE of 0.675, suggesting its strong ability to identify patterns in stockout trends over the years. This multi-method technique, summarized in Table 4, provides a solid foundation for identifying causal relationships and improving predictability in supply chain stockout risk management. Furthermore, to construct a baseline, we employed a typical Linear Regression approach to stockout risk prediction. The regression model recorded a low R^2^ of 0.00063, meaning it describes almost no variation in the dependent variable, and an extremely high Mean Squared Error (MSE) of 18.89. Our ensemble approach, Random Forest and LightGBM, attained R^2^ values of 0.25 and lower MSEs of 0.736–0.741, indicating significant increase in predicting ability. This contrast demonstrates that the proposed ensemble paradigm outperforms simple linear methods for modelling complex supply chain behaviors.

In addition, IV causal inference approach, yielded an Average Treatment Effect (ATE) of 0.5535 having a p-value of 0.3148, suggesting the impact assessment wasn’t statistically noteworthy at standard thresholds. The approach’s R^2^ score of 0.2435 indicates moderate explanation power. In comparison with other simulations, the IV algorithm’s R^2^ is slightly lesser compared to the RF and LGBM approaches, which both attained R^2^ scores of 0.25, and significantly lower than the time series approach Moving Average, that attained the highest R^2^ of 0.883. The high R^2^ reveals the robustness of the persistence and autocorrelation in the simulated stockout process, instead of the model complexity, reflecting the controlled simulation setup.

An ATE of 0.882 shows that persistent lead time increases the stockout risk index by approximately 0.882 units on a 0–1 scale proportional to shorter lead times, under the assumed conditions.

**Fig 8:**
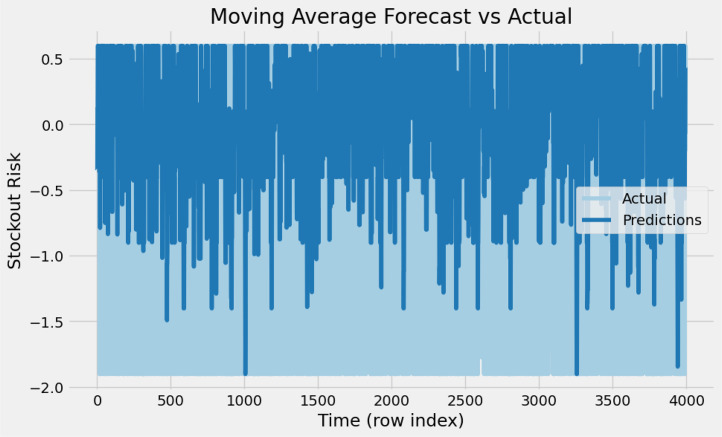
Moving average forecast.

Fig 8 compares the expected stockout risk to the actual values over an extended period. The dark blue line indicates the projections, whereas the less colored area shows the actual stockout risk levels. The plot shows that the moving average approach covers overall stockout risk patterns, but there are considerable variances, particularly during strong oscillations. The model accurately predicts stockout risk over the years, as evidenced by the high R^2^ of 0.883 and low MAE of 0.675 based on experimental data.

To prevent data leakage and to maintain temporal validity, the MA technique was evaluated using a rolling origin forecasting procedure. During each predictions step, the MA window integrated only historical observations based exclusively on information available prior to the forecast horizon. No information from future time points was used in model development or evaluation.

This research identifies practical factors for supply chain risk management, which enables it to provide practical consequences at both the operational and policy levels. Thus, due to the fact that the outcomes of the causative investigation establish lead time as an intervening variable, it is recommended that organizations increase their supplier procurement guidelines, logistics organization, and lead-time reserve contracts as architectural risk control process. Therefore, in order to facilitate flexible procurement and performance-driven supplier management, predictive modelling provides the capability to foresee risks in the short term. On the other hand, temporal analysis suggests that safety stock programs should reflect periodic and lasting risk trends rather than depending on fixed reserve levels. As a whole, these realizations lend support to the creation of unified risk monitoring systems and situational planning techniques. They also demonstrate how linked management, forecasting, and monitoring can improve the robustness of supply chain operations.

## 5. Conclusion

The study offers an innovative, multi-method paradigm that combines causal inference and predictive analytics to tackle the long-standing problem of stockout risk in supply chain management. Unlike previous research that concentrated solely on ML prediction or optimization methods, our approach combines causal inference methods with ML techniques and time series forecasting, providing both predictive and explanatory ability.

In comparison with [19], who employed stochastic programming and ML for outbreak related healthcare supply chains, our study is more generalizable and interpretable by calculating the causal influence of lead time on stockout risk in wider supply chain settings. While they focused on resilience in unpredictability, the application of estimates of causality (ATE) and real-time predictive algorithm provides more supervisory knowledge for day-to-day operational strategy.

Similarly, [16] created a forecasting warning system to predict warehouse cycle time, whereas [4] improved backorder prediction using basic ML models. However, neither investigated the fundamental causes of supply chain interruptions. Our study fills this gap by giving measurable proof of how decreasing lead time causally influences stockout probability, hence improving decision-making across forecasting.

Furthermore, studies by [2,15] emphasized the use of AI/ML to predict inefficiencies and maintenance. Although methodologically correct they are mostly predictive and lack causative tools for diagnosis. In contrast, our hybrid technique predicts risk while also assisting in the identification and quantification of controlled levers, such as lead time, which may mitigate it.

In conclusion, this study advances to the body of existing literature by employing strong econometric techniques to quantify the causal relationship between lead time and stockouts. Hence, u sing ensemble ML algorithms to get high predicting performance. Also, establishing a connection between prediction. Supplying supply chain managers with doable tactics to prevent interruptions in the first place. These contributions show that, in addition to being innovative, our integrated technique is practically better at facilitating data-driven, causally influenced inventories decisions, a feature that is mainly lacking in previous research.

Despite the fact that this study offers a strong multidisciplinary approach that combines time-series forecasting, ML, and causal inference, it has some constraints. Novel approaches like DML or CATE assessment for diverse impacts were not extensively investigated despite the application of important causal inference methods. However, the absence of DML in the study is not detrimental from the current study’s authenticity or uniqueness. We used a variety of well-established methods to identify causality, each with its own set of assumptions and estimating strategies. The convergence of data from PSM and IPW, both of which produced substantial and significant ATE projections, supports the validity of our results. In addition, despite its limitations, our use of IV analysis provides insight into instrument validity and encourages further technical development.

## Supporting information

S1 Data Synthetic supply chain data. (CSV)
